# The HLA ligandome of oropharyngeal squamous cell carcinomas reveals shared tumour-exclusive peptides for semi-personalised vaccination

**DOI:** 10.1038/s41416-023-02197-y

**Published:** 2023-02-23

**Authors:** Lena Mühlenbruch, Tsima Abou-Kors, Marissa L. Dubbelaar, Leon Bichmann, Oliver Kohlbacher, Martin Bens, Jaya Thomas, Jasmin Ezić, Johann M. Kraus, Hans A. Kestler, Adrian von Witzleben, Joannis Mytilineos, Daniel Fürst, Daphne Engelhardt, Johannes Doescher, Jens Greve, Patrick J. Schuler, Marie-Nicole Theodoraki, Cornelia Brunner, Thomas K. Hoffmann, Hans-Georg Rammensee, Juliane S. Walz, Simon Laban

**Affiliations:** 1 Institute for Cell Biology, Department of Immunology, Eberhard Karls University of Tübingen, 72076 Tübingen, Baden-Württemberg Germany; 2 Department of Peptide-based Immunotherapy, Eberhard Karls University and University Hospital Tübingen, 72076 Tübingen, Baden-Württemberg Germany; 3 Cluster of Excellence iFIT (EXC2180) “Image-Guided and Functionally Instructed Tumor Therapies”, University of Tübingen, 72076 Tübingen, Baden-Württemberg Germany; 4 German Cancer Consortium (DKTK), Partner Site Tübingen, 72076 Tübingen, Baden-Württemberg Germany; 5 Department of Otorhinolaryngology and Head & Neck Surgery, Ulm University Medical Center, Head and Neck Cancer Center of the Comprehensive Cancer Center Ulm, Ulm, Germany; 6 Quantitative Biology Center (QBiC), Eberhard Karls University Tübingen, 72076 Tübingen, Baden-Württemberg Germany; 7 Applied Bioinformatics, Department of Computer Science, Eberhard Karls University Tübingen, 72076 Tübingen, Baden-Württemberg Germany; 8 Cluster of Excellence Machine Learning in the Sciences (EXC2064), Eberhard Karls University Tübingen, 72076 Tübingen, Baden-Württemberg Germany; 9 Institute for Translational Bioinformatics, University Hospital Tübingen, 72076 Tübingen, Baden-Württemberg Germany; 10 Institute for Bioinformatics and Medical Informatics, Eberhard Karls University Tübingen, 72076 Tübingen, Baden-Württemberg Germany; 11 Leibniz-Institute on Aging, Fritz-Lipmann-Institute, 07745 Jena, Thüringen Germany; 12 CRUK and NIHR Experimental Cancer Medicine Center & School of Cancer Sciences, Faculty of Medicine, University of Southampton, Southampton, SO17 1BJ UK; 13 Ulm University, Institute of Medical Systems Biology, Ulm, Germany; 14 Institute of Clinical Transfusion Medicine and Immunogenetics Ulm, German Red Cross Blood Transfusion Service, Baden–Württemberg–Hessen, and University Hospital Ulm, Ulm, Germany; 15 Institute of Transfusion Medicine, Ulm University, Ulm, Germany; 16 German Stem Cell Donor Registry, German Red Cross Blood Transfusion Service, Ulm, Germany; 17 Clinical Collaboration Unit Translational Immunology, German Cancer Consortium (DKTK), Department of Internal Medicine, University Hospital Tübingen, Tübingen, Baden-Württemberg 72076 Germany

**Keywords:** Tumour immunology, Translational research

## Abstract

**Background:**

The immune peptidome of OPSCC has not previously been studied. Cancer-antigen specific vaccination may improve clinical outcome and efficacy of immune checkpoint inhibitors such as PD1/PD-L1 antibodies.

**Methods:**

Mapping of the OPSCC HLA ligandome was performed by mass spectrometry (MS) based analysis of naturally presented HLA ligands isolated from tumour tissue samples (*n* = 40) using immunoaffinity purification. The cohort included 22 HPV-positive (primarily HPV-16) and 18 HPV-negative samples. A benign reference dataset comprised of the HLA ligandomes of benign haematological and tissue datasets was used to identify tumour-associated antigens.

**Results:**

MS analysis led to the identification of naturally HLA-presented peptides in OPSCC tumour tissue. In total, 22,769 peptides from 9485 source proteins were detected on HLA class I. For HLA class II, 15,203 peptides from 4634 source proteins were discovered. By comparative profiling against the benign HLA ligandomic datasets, 29 OPSCC-associated HLA class I ligands covering 11 different HLA allotypes and nine HLA class II ligands were selected to create a peptide warehouse.

**Conclusion:**

Tumour-associated peptides are HLA-presented on the cell surfaces of OPSCCs. The established warehouse of OPSCC-associated peptides can be used for downstream immunogenicity testing and peptide-based immunotherapy in (semi)personalised strategies.

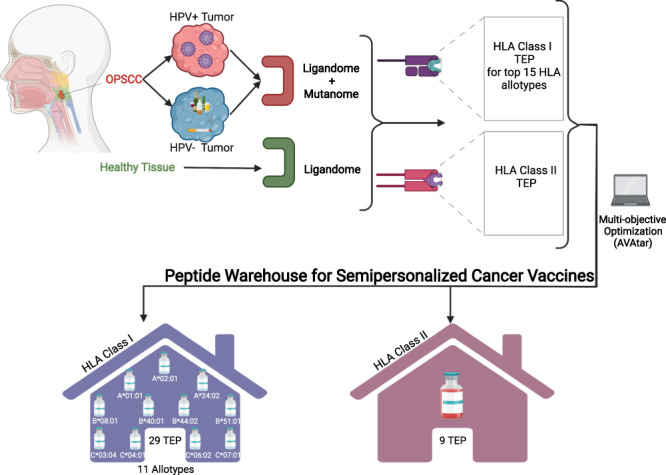

## Background

Oropharyngeal squamous cell carcinoma (OPSCC) is diagnosed in 93,000 patients worldwide per year and 51,000 annual deaths can be attributed to this disease [1]. In OPSCC, human papillomavirus induced (HPV-positive) cancers and non-virally associated, primarily tobacco- and alcohol-associated (HPV-negative) cancers must be discriminated [2, 3]. For different types of curative treatment, a survival advantage for HPV-positive OPSCC has been confirmed [4–6]. As a result, the latest classification of the American Joint Committee on Cancer (AJCC) cancer staging manual version 8 discriminates between HPV-positive and HPV-negative cancers based on the surrogate marker p16 [7, 8]. Immunotherapy targeting the PD1/PD-L1 axis has become a central column of treatment in recurrent and metastatic disease [9–11] and is currently studied intensively in locoregionally advanced disease [12–14]. In recurrent and metastatic disease, the response rates of anti-PD1 antibodies lie below 20% [9–11]. Because the success of PD1/PD-L1 antibodies relies on the presence of pre-existing cancer-antigen specific immunity [15], vaccination against cancer antigens may improve efficacy of such treatments [16]. However, it is currently unclear which antigens should be targeted. Immune responses to viral antigens in HPV-positive disease [17, 18] and immune responses to other cancer antigens [19, 20] including mutation-associated antigens, so called neoantigens, have previously been described [21, 22]. The respective significance of the different types of cancer antigens for immunotherapy is currently unclear.

To establish vaccination strategies for OPSCC, it is crucial to understand its antigenic landscape. Tumour-specific immune cells rely on the presentation of peptides from cancer antigens on human leucocyte antigens (HLA)—the immunopeptidome or HLA ligandome. For optimal immune responses against the tumour, these HLA-presented peptides need to be tumour-exclusive. Thus, the analysis of the HLA ligandome can be used to identify promising disease-specific vaccination targets [23–26].

Here we performed the first comprehensive analysis of the natural HLA ligandome of OPSCC by mass spectrometry to guide personalised or semi-personalised vaccine development.

## Materials and methods

### Patients

Patients with histologically confirmed OPSCC who were treated surgically were included into this non-interventional study except for one patient who preferred definitive chemoradiotherapy. The sample was taken during panendoscopy in this patient. Fresh frozen tissue samples derived from 22 HPV-positive and 18 HPV-negative patients were prospectively collected at Ulm University Medical Center before treatment initiation. HPV status was determined via RNA-Seq (compare below).

Among the 22 HPV-positive patients, 19 were associated with HPV-16 and the other three with HPV-35, HPV-58 or HPV-59, respectively. OPSCC tumour biopsies and tonsillar tissue samples from five healthy donors were surgically resected, immediately snap-frozen in liquid nitrogen (N_2_) and subsequently stored at −80 °C. Written informed consent was obtained in accordance with the Declaration of Helsinki protocol. The study was performed according to the guidelines of the local ethics committee (222/13, 90/15). Patient demographics including sex are provided in Table 1.

**Table 1:** Patient characteristics.

		HPV negative	%	HPV positive	%	Total	%
Total		18	45.0%	22	55.0%	40	100.0%
Sex	Male	12	66.7%	21	95.5%	33	82.5%
	Female	6	33.3%	1	4.5%	7	17.5%
Age		59.6	n.a.	60.2	n.a.	59.9	n.a.
pT^a^	pT1/T2	7	38.9%	11	50.0%	18	45.0%
	pT3/T4	10	55.6%	11	50.0%	21	52.5%
	Missing	1	5.6%	0	0.0%	1	2.5%
pN^a^	pN0	6	33.3%	8	36.4%	14	35.0%
	pN1	4	22.2%	6	27.3%	10	25.0%
	pN2	2	11.1%	7	31.8%	9	22.5%
	pN3a/b	5	27.8%	1	4.5%	6	15.0%
	Missing	1	5.6%	0	0.0%	1	2.5%
Stage (UICC v8)	I	1	5.6%	7	31.8%	8	20.0%
	II	3	16.7%	10	45.5%	13	32.5%
	III	6	33.3%	3	13.6%	9	22.5%
	IV (a/b)	8	44.4%	2	9.1%	10	25.0%
R status^a^	R0	13	72.2%	20	90.9%	33	82.5%
	R1	1	5.6%	1	4.5%	2	5.0%
	Close margin (<5 mm)	3	16.7%	1	4.5%	4	10.0%
L status^a^	L0	9	50.0%	12	54.5%	21	52.5%
	L1	8	44.4%	10	45.5%	18	45.0%
	Missing	1	5.6%	0	0.0%	1	2.5%
V status^a^	V0	17	94.4%	20	90.9%	37	92.5%
	V1	0	0.0%	2	9.1%	2	5.0%
	Missing	1	5.6%	0	0.0%	1	2.5%
Pn status^a^	Pn0	11	61.1%	20	90.9%	31	77.5%
	Pn1	6	33.3%	2	9.1%	8	20.0%
	Missing	1	5.6%	0	0.0%	1	2.5%
ENE^a^	ENE-negative	5	27.8%	3	13.6%	8	20.0%
	ENE-positive	6	33.3%	10	45.5%	16	40.0%
Grading	Well differentiated	1	5.6%	0	0.0%	1	2.5%
	Moderately differentiated	8	44.4%	6	27.3%	14	35.0%
	Poorly differentiated	9	50.0%	16	72.7%	25	62.5%
	Undifferentiated	0	0.0%	0	0.0%	0	0.0%
Subsite	BOT	4	22.2%	1	4.5%	5	12.5%
	Soft palate	1	5.6%	0	0.0%	1	2.5%
	Tonsil	13	72.2%	21	95.5%	34	85.0%
Smoking history	Never smoker	1	5.6%	3	13.6%	4	10.0%
	Former smoker	6	33.3%	12	54.5%	18	45.0%
	Current smoker	11	61.1%	6	27.3%	17	42.5%
	Missing	0	0.0%	1	4.5%	1	2.5%
	Pack years (mean)	30	n.a.	20	n.a.	24.8	n.a.
Alcohol consumption	Never	0	0.0%	1	4.5%	1	2.5%
	Sometimes	5	27.8%	14	63.6%	19	47.5%
	Daily	8	44.4%	4	18.2%	12	30.0%
	Intensively daily	2	11.1%	0	0.0%	2	5.0%
	Former heavy drinker	3	16.7%	2	9.1%	5	12.5%
	Former consumption	0	0.0%	0	0.0%	0	0.0%
	# Drinks per week (mean)	32	n.a.	23	n.a.	30	n.a.

*pT* pathological primary tumour classification, *pN* pathological nodal classification, *R* resection status, *L* lympangioinvasion, *V* vascular invasion, *Pn* perineural invasion, *HPV* human papillomavirus, *ENE* extranodal extension, *BOT* base of tongue.

^a^One patient did not receive a surgical resection, but preferred primary chemoradiation.

### HLA typing

High resolution HLA-typing for the loci HLA-A, -B, -C, -DRB1, -DQB1, and -DPB1 was performed using an in-house developed IVD-CE-certified NGS-amplicon sequencing protocol (H-Seq-LR-NGS, DRK-BSD, Baden-Württemberg Hessen, Ulm), based on the Illumina MiSeq platform (San Diego, CA, USA) [27] or based on the HLA-binding motifs of isolated HLA-presented peptides using SYFPEITHI [28] and NetMHC [29].

### Isolation of HLA-presented peptides

HLA class I- and class II-presented peptides were isolated from tissue samples performing standard immunoaffinity purification as previously described [30]. The HLA class I A-, B-, and C-specific monoclonal antibody (mAb) W6/32, the pan-HLA class II-specific mAb Tü-39, and the HLA-DR-specific mAb L243 (all produced in-house) were used to extract HLA molecules.

### Analysis of HLA ligands by liquid chromatography–tandem MS

HLA ligand extracts were analysed as previously described [25] and were separated using reversed-phase ultra-high performance liquid chromatography (nanoUHPLC, UltiMate 3000 RSLCnano, Dionex). Eluted peptides were analysed by tandem mass spectrometry (MS/MS) in an on-line coupled LTQ Orbitrap XL hybrid mass spectrometer (Thermo Fisher Scientific) equipped with a nano-electrospray ion source.

### Data processing

Processing of MS data was performed using the Proteome Discoverer 1.4 software (Thermo Fisher Scientific). Database search and spectral annotation were performed against the human proteome as comprised in the UniProtKB/Swiss-Prot database (20,279 reviewed protein sequences; September 27, 2013; www.uniprot.org) via the SequestHT algorithm. Search for HLA-presented peptides derived from viral proteins was based on the HPV proteome as comprised in the UniProtKB/Swiss-Prot database (470 reviewed protein sequences; January 5, 2018; www.uniprot.org). Mass tolerance for processing was set to 5 ppm for precursor ions and 0.5 Da for fragment ions. Oxidised methionine was allowed as only dynamic modification and no cleavage specificity was selected. Peptide identifications were filtered using the Percolator 2.04 [31] with a target value of *q* ≤ 0.05 (5% FDR). Additional filters for search engine rank (=1) and peptide length (=8–25 amino acids) were applied. HLA class I ligand annotation was performed using SYFPEITHI [28] and NetMHCpan 4.0 [29].

### HPV typing/RNA sequencing

For this analysis, RNA sequencing data were only used to define HPV status. Total RNA was extracted using AllPrep DNA/RNA Mini Kit (Qiagen, Germany) from fresh, snap-frozen tumour samples. Sequencing of RNA samples was performed using Illumina’s next-generation sequencing methodology [32]. In detail, total RNA was quantified and quality checked using Agilent 2100 Bioanalyzer Instrument (Agilent RNA 6000 Pico). Libraries were prepared from 500 ng of input material using TruSeq Stranded mRNA (manufacturer’s instructions) and subsequently quantified and quality checked using Agilent 2100 Bioanalyzer Instrument (DNA 7500 kit). Libraries were pooled and sequenced in one lane of HiSeq 2500 System running in 51 cycle/single-end/high output mode. Sequence information was converted to FASTQ format using bcl2fastq (2.20.0.422). High-quality SE reads were mapped to the human genome (hg38) using STAR (2.0.9) and, following the removal of multimapping reads, converted to gene-specific read counts for annotated genes using featureCounts (2.0.0). Unmapped reads to the human genome were aligned to HPV high-risk type genomes using a viGen bioinformatic pipeline [33]. Samples with ≥500 reads for HPV E6 or E7 RNA or ≥500 reads for all HPV oncogenes (E1, E2, E4, E5, E6, E7, L1, L2) in summary were considered HPV-positive. The HPV type with the highest number of reads was selected. HPV-negative cases had a mean of 8 reads for E1, E2, E4, E5, E6, E7, L1, L2 in summary (range: 0–23). HPV RNA reads for HPV-16, -18, -35, -58, and -59 are shown in supplementary table 1. In addition to RNA sequencing, data for p16 immunohistochemistry and HPV DNA PCR were available for all samples. Our complete HLA-peptidome dataset was specifically queried for HPV-specific peptides.

### Whole-exome sequencing

DNA was extracted using the Qiagen AllPrep DNA/RNA Mini Kit. Sequencing of exome samples was performed using Illumina’s next-generation sequencing methodology [32]. In detail, total DNA was quality checked using Agilent 4200 TapeStation System (Agilent Genomic DNA ScreenTape) and quantified using Quant-iT™ PicoGreen™. Libraries were prepared from 3 µg of input material using SureSelect Human All Exon V6 (manufacturer’s instructions) and subsequently quantified and quality checked using Agilent 4200 TapeStation System (D1000 ScreenTape). Libraries were pooled and sequenced on NextSeq 500 System (High Output Flow Cell) running in 150 cycle (2 × 75 bp paired-end) mode. Sequence information was converted to FASTQ format using bcl2fastq v2.20.0.422. The WES data were used to search for HLA-presented individual neoepitopes in the complete OPSCC HLA ligandome dataset of each patient with available whole exome sequencing data (38/40 patients). Database search and spectral annotation were performed against the combination of the human proteome as comprised in the UniProtKB/Swiss-Prot database and the mutated protein sequences as defined for the respective patients.

### Software, statistical analysis and online tools

For overlap analysis, BioVenn [34] and jVenn [35] were used. The benign reference dataset used for comparative profiling was comprised of the HLA ligandome data of a previously reported haematological benign cohort [36], the benign tissue dataset provided within the HLA Ligand Atlas [37] as well as additional in-house acquired HLA ligandome data of benign tissue and cell line samples. HLA ligandome data were also compared to previously published immunopeptidomes of other solid malignant diseases (ovarian cancer [25], hepatocellular carcinoma [38], renal cell carcinoma [39], glioblastoma [40]). Statistical analysis was performed using the GraphPad Prism 6.1/9.01 software (GraphPad Software Inc).

The software AVAtar, previously developed at Ulm University [41], was used to determine peptide combinations for the Top 5 HLA class I allotypes of HLA-A, -B and -C using multiobjective optimisation of coverage for the respective HLA allotype and the number of antigens selected as previously described [41]. Tumour-exclusive peptides (TEP) found in ≥2 patients of a certain class I allotype were filtered. These peptide candidates were subjected to multiobjective optimisation with preset configurations and 1 × 10^6^ iterations for the HLA allotypes with the highest prevalence in the cohort. Each peptide that appeared in a selection underwent additional quality control (QC) to ensure sufficient goodness and specificity of fit between experimental and theoretical spectra. This entailed minimum requirements for the number of peptide spectrum matches (PSMs ≥2), the cross-correlation value (Xcorr ≥1.5) and the delta correlation score between primary and the secondary sequence candidates (ΔCn ≥0.2). All peptides appearing during the optimisation runs were considered potential candidates for a peptide warehouse. In total, up to 3 optimisation runs were performed if peptides in the selection had to be removed after QC.

## Results

### The HLA ligandome of oropharyngeal squamous cell carcinomas (OPSCCs)

The OPSCC patient study cohort (*n* = 40) comprised a total of 49 different HLA class I allotypes, covering at least one HLA class I allotype for 99.93% of individuals within the world’s population (Supplementary Fig. 1A) [42, 43]. Most frequent alleles within the patient cohort were HLA-A*02:01 (*n* = 25), HLA-A*01:01 (*n* = 15), HLA-C*07:01 (*n* = 14), HLA-C*04:01 (*n* = 13) and HLA-B*51:01 (*n* = 12). For each class I Isotype (HLA-A, -B, -C), the top 5 alleles were highlighted in Supplementary Fig. 2A–C and used for multiobjective optimisation (compare below). The distribution of HLA class I allele frequencies was representative for most alleles in comparison to a German reference population (cohort “Germany pop 8” (*n* = 39,689); www.allelefrequencies.net) [44]. In contrast, the allele frequency of HLA-B*51:01 was significantly higher in OPSCC patients compared to the reference cohort, respectively (16.2% and 6.3%; *p* = 0.0005; OR = 2.9; 95% CI = 1.6–5.3) (Supplementary Fig. 2A–C).

**Fig. 1:**
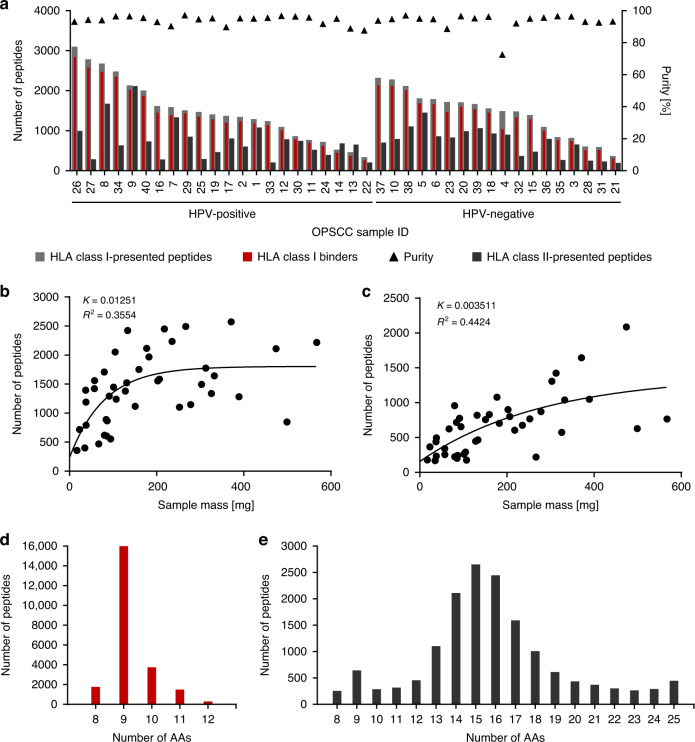
HLA ligand and sample characteristics. **a** Yields of isolated HLA class I- and class II-presented peptides for individual OPSCC samples achieved by LC-MS/MS analysis. Peptide yields varied between 265 and 2,854 HLA class I-presented (mean = 1424) and between 168 and 2086 HLA class II-presented peptides (mean = 702) per individual sample. HLA class I ligands were defined as HLA class I-presented peptides carrying a binding motif of an HLA allotype of the respective patient using SYFPEITHI (Rammensee et al. [28]) and NetMHCpan 4.0 (Andreatta and Nielsen [29]). The purity is defined as the proportion of binders among all HLA class I-presented peptides and indicated by black triangles on the right *y*-axis. Correlation of sample masses and yields of **b** HLA class I ligands and **c** HLA class II-presented peptides. Length distribution analysis of **d** HLA class I ligands and **e** HLA class II-presented peptides. OPSCC oropharyngeal squamous cell carcinoma; *n*(OPSCCs) = 40; AA amino acid.

LC-MS/MS-based analysis of the HLA class I ligandomes isolated from 40 OPSCC tissue samples identified a total of 22,769 unique HLA class I ligands (range: 265–2854 peptides per sample; mean: 1338 peptides per sample) derived from 9485 different source proteins (Fig. 1a and Supplementary Table 2) and, thereby, obtaining 98% of the estimated maximum attainable coverage in HLA ligand source proteins (Supplementary Fig. 1C). A weak positive correlation between tissue sample masses and yields of HLA class I ligands was observed (*p* = 0.0019; Pearson’s correlation coefficient *r* = 0.4645; 95% confidence interval (CI) = 0.2–0.7) (Fig. 1b). As expected, the majority of HLA class I ligands (70%) exhibited a peptide length of 9 amino acids (Fig. 1d).

Thirty-three different HLA-DRB, 13 different HLA-DQB and 12 different HLA-DPB allotypes were comprised in the OPSCC patient cohort, covering at least one HLA class II allotype in 99.99% of individuals among the world’s population (Supplementary Fig. 1B) [42, 43]. The most frequent alleles within the OPSCC cohort were HLA-DPB1*04:01 (*n* = 23), HLA-DQB1*03:01 (*n* = 17), HLA-DRB4*01:03 (*n* = 13) and HLA DPB1*03:01 (*n* = 12) in descending order. The comparison of the HLA-DRB1 allelic prevalence in the OPSCC patient cohort and a German reference population (cohort “Germany pop 8” (*n* = 39,689), www.allelefrequencies.net) revealed no significant differences in HLA class II allelic distribution (Supplementary Fig. 2D) [44].

Mapping the HLA class II ligandomes of 40 OPSCC tissue samples by LC-MS/MS revealed a total of 15,203 unique HLA class II-presented peptides (range: 168–2086 peptides per sample; mean: 702 peptides per sample) from 4634 source proteins (Fig. 1a and Supplementary Table 2), achieving a 74% coverage of the estimated maximum attainable number of source proteins (Supplementary Fig. 1D). A positive correlation of sample masses and yields of HLA class II-presented peptides was shown (*p* < 0.0001; Pearson´s correlation coefficient *r* = 0.6388; 95% CI = 0.4–0.8) (Fig. 1c). Lengths of HLA class II-presented peptides were distributed across the tolerated range of 8–25 amino acids, with 15 amino acids as the most abundant peptide length (17%) (Fig. 1e).

### OPSCC-associated HLA ligands

**Fig. 2:**
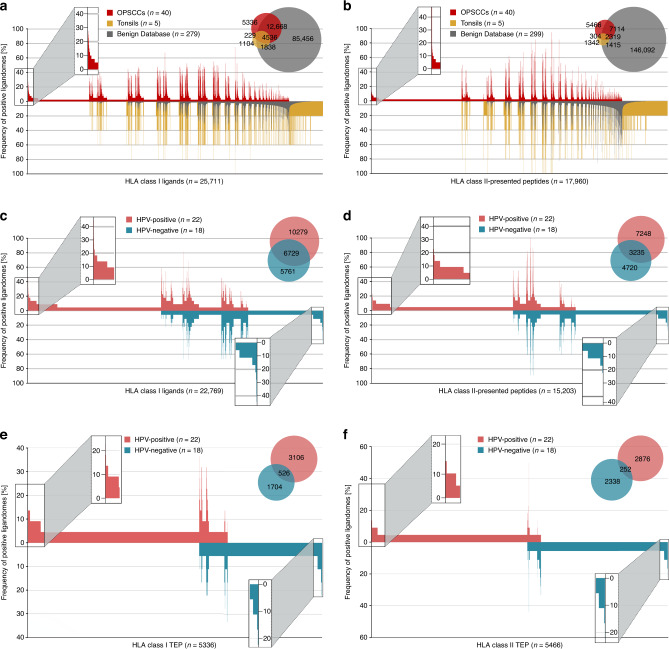
Tumour-exclusive Peptides (TEP) in OPSCC ligandomes. Comparative profiling of **a** HLA class I ligands and **b** HLA class II-presented peptides based on the frequency of presentation in OPSCC and benign ligandomes. Comparative profiling of **c** HLA class I ligands and **d** HLA class II-presented peptides based on the frequency of presentation in HPV-positive and HPV-negative OPSCC ligandomes. Comparative profiling of **e** HLA class I TEP and **f** HLA class II TEP based on the frequency of presentation in HPV-positive and HPV-negative OPSCC ligandomes. Frequencies of positive immunopeptidomes for the respective peptides (*x*-axis) are indicated on the *y*-axis.

To identify OPSCC-associated antigens, comparative HLA class I and class II ligandome profiling of the OPSCC cohort was performed against a benign reference dataset. This dataset mainly encompassed HLA ligandome data of a previously reported haematological benign cohort [36], the benign tissue dataset provided within the HLA Ligand Atlas [37] as well as a newly established tonsillar HLA ligandome dataset from five healthy control samples (Supplementary Table 3). Together, the benign reference database contained HLA class I ligandome data from 35 haematological and non-haematological tissue types (*n* = 424 samples) comprising a total of 153,733 unique HLA class I-presented peptides derived from 17,200 different source proteins. Overlap analysis between the OPSCC and the benign reference datasets revealed 5336 HLA class I ligands presented exclusively on OPSCC samples (Fig. 2a). 101 of these tumour-exclusive peptides (TEP) were identified with a prevalence of ≥7.5 (≥3 samples) among the OPSCC patients. Three thousand two hundred and fifty-one TEP were newly identified peptides not present in our previously published solid tumour immunopeptidomes (ovarian cancer [25], hepatocellular carcinoma [38], renal cell carcinoma [39], glioblastoma [40]).

Regarding the HLA class II ligandomes, the benign reference database was comprised of immunopeptidomic data from 33 haematological and non-haematological tissue types (*n* = 369 samples) with a total of 156,940 unique HLA class II-presented peptides derived from 16,035 source proteins. Overlap analysis revealed 5466 OPSCC-exclusive HLA class II-presented peptides (Fig. 2b). Eighty-two of these TEP were identified with a prevalence of ≥7.5% (≥3 samples) among patients. Four thousand eight hundred and thirty-seven TEP were newly identified peptides that were not discovered in our previously published solid tumour immunopeptidomes (ovarian cancer [25], renal cell carcinoma [39], glioblastoma [40]).

### HPV-associated HLA ligands

The present cohort contained 40 tumour samples originating from 22 HPV-positive and 18 HPV-negative patients. In none of the 40 OPSCC immunopeptidomes, HLA class I binders or HLA class II-presented peptides were detected that derived from an HPV source protein or from mutated neoantigens.

However, a supervised principal component analysis (PCA) and corresponding heatmap based on the merged source proteins of HLA class I ligands and of HLA class II-presented peptides resulted in a clear separation of the samples into HPV-positive and HPV-negative tumours (Supplementary Fig. 3). HLA class I binders and HLA class II-presented peptides were identified that were either shared or exclusively presented by HPV-positive or HPV-negative OPSCC samples (Fig. 2c, d). Comparative analysis revealed 10,279 HLA class I ligands exclusive for HPV-positive and 5761 HLA class I ligands exclusive HPV-negative OPSCCs derived from 5952 and 4231 source proteins, respectively. Among these, 653 HLA class I ligands exclusive for HPV-positive and 190 HLA class I ligands exclusive for HPV-negative OPSCCs were identified in ≥3 samples of the respective subgroup.

Seven thousand two hundred and forty-eight HLA class II-presented peptides exclusive for HPV-positive and 4720 exclusive for HPV-negative OPSCCs were identified derived from 2859 and 2633 source proteins, respectively. Of these, 197 peptides exclusive for HPV-positive and 88 peptides exclusive for HPV-negative OPSCCs were detected in ≥3 samples of the respective subgroups.

These results indicate that immunopeptidomes of OPSCCs differ in their composition of antigens depending on the patients’ HPV status. This also applies to TEP as shown in Fig. 2e, f.

**Fig. 3:**
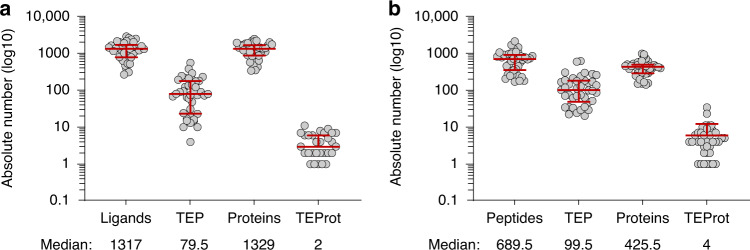
Frequency distribution of HLA ligands, tumour-exclusive peptides, source proteins and tumor-exclusive proteins. Absolute numbers of HLA ligands or -presented peptides, tumour-exclusive (TEP) peptides, source proteins and tumour-exclusive proteins (TEProt) for **a** HLA class I and **b** HLA class II on a logarithmic scale (log10). Red lines represent the median and interquartile range. The median values are indicated below the *x*-axis labels.

There were no significant differences in the number of total and TEP or total and tumour-exclusive proteins per patient compared by HPV status. The median number of HLA class I binders per patient and TEP identified was 1317 (range: 265–2854) and 79.5 (range: 4–552), respectively, and the median number of HLA class I ligand source proteins per patient and tumour-exclusive source proteins was 1329 (range: 338–2444) and 2 (range: 0–11), respectively. Medians with interquartile range are graphed in Fig. 3a.

The median number of HLA class II peptides per patient and TEP identified was 689.5 (range: 168–2086) and 99.5 (range: 20–608), respectively, and the median number of source proteins of HLA class II-presented peptides per patient and tumour-exclusive source proteins was 425.5 (range: 139–959) and 4 (range: 0–34), respectively. Medians with interquartile range are graphed in Fig. 3b.

### Establishment of OPSCC peptide warehouse

Multiobjective optimisation employing AVAtar [41] software was used to uncover selections of HLA class I TEP by HLA class I allotype with maximal coverage and a minimal number of peptides for the above mentioned 15 HLA allotypes.

**Fig. 4:**
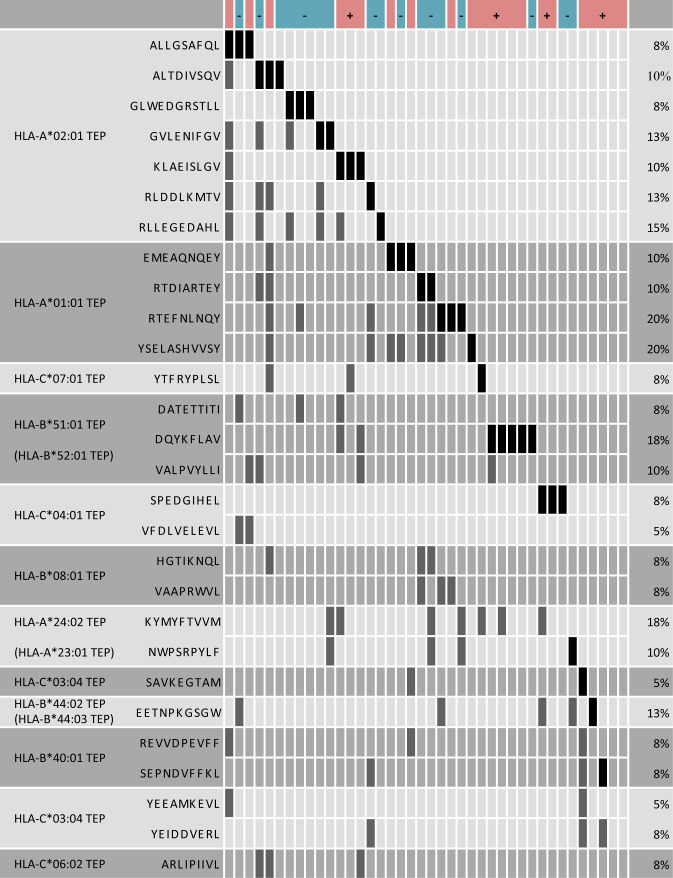
Warehouse of TEP for 11 HLA allotypes. The HLA allotype is indicated in the left column, the next column shows the peptide amino acid sequence. Patients are presented in columns and peptide coverage is indicated by dark bars. Black bars indicate samples that contribute directly to coverage, whereas overlap is indicated by grey bars. Patient HPV status is indicated in the top line (red—HPV-positive (+), blue—HPV-negative (−)). The right column indicates peptide prevalence in relation to the whole cohort (*n* = 40). The graph was generated using AVAtar (Völkel et al. [41]).

Central data from the optimisation and selection process are shown in Supplementary Table 4. In total, 29 TEP were selected for 11 HLA allotypes. For the remaining four allotypes, contribution to coverage was negligible due to a low number of patients presenting the same TEP. The resulting selections of TEP for each of the most frequent allotypes and the respective source proteins are shown in Fig. 4 and Supplementary Table 5. Only 2 HPV-positive patients did not present any of the 29 TEP. The merged selections by HPV status are shown in Supplementary Fig. 4.

Multiobjective optimisation was also performed for HLA class II TEP to identify a selection of TEP with maximal coverage using a minimal number of peptides. From 71 HLA class II TEP that were found in ≥3 patients, 57 peptides were eliminated during quality control. Among the 14 HLA class II TEP, a selection of 9 TEP resulted in the maximal coverage of 62.5% for all patients (HPV-negative: 50%, HPV-positive: 72.7%). This selection and its coverage by HPV status is shown in Supplementary Fig. 5.

**Fig. 5:**
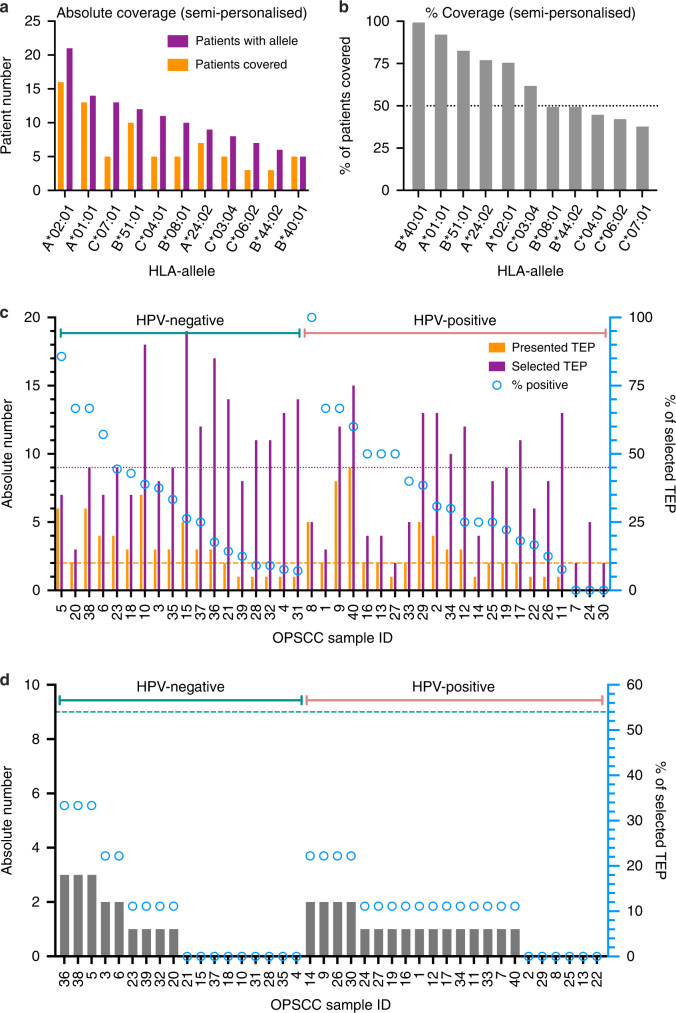
Patient coverage by semi-personalised warehouse peptide selections. **a** Bar graph indicating the absolute number of patients with the respective HLA allele (purple) and patients presenting at least one of the semi-personalised peptides selected (orange) ordered by the prevalence of the respective HLA allele in the cohort. **b** Bar graph showing the relative number of patients covered by at least one peptide of the semi-personalised peptide selection ordered from highest coverage to lowest. **c** Bar graph indicating the absolute number of HLA class I peptides selected (purple) for the semi-personalised TEP selection for each individual patient based on its HLA typing and the absolute number of actually presented peptides selected (orange) graphed on the left *y*-axis. The right *y*-axis displays the relative number of presented peptides in relation to the number of peptides selected (% of selected TEP, blue circles). Patients were ordered by HPV-status and descending coverage. **d** Bar graph showing the absolute number of HLA-class II peptides presented per patient from the selection on the left *y*-axis. Blue circles indicate the coverage (%, right *y*-axis) in relation to the 9 selected HLA class II TEP (pointed horizontal line).

Absolute and relative coverage of patients for each of the 11 HLA class I allotype specific TEP are shown in Fig. 5a, b, respectively. The selections resulted in a coverage ranging from 100% for HLA-B*40:01 to 38.5% for HLA-C*07:01. The allotype-specific selections covered ≥50% of patients with the respective allele for HLA-B*40:01, HLA-A*01:01, HLA-B*51:01, HLA-A*24:02, HLA-A*02:01 HLA-B*08:01 and HLA-B*44:02.

Next, a semi-personalised combination of HLA class I TEP was selected for every patient based on the individual HLA typing results to mimic a theoretical semi-personalised vaccine composition. These semi-personalised HLA class I TEP combinations are shown in Fig. 5c and resulted in a median of 9 selected HLA class I TEP per patient (range: 2–19). A median of 2 HLA class I TEP among the semi-personalised selected TEP were presented in the respective patient’s ligandome (range: 0–9), resulting in a median coverage of 28.14% of the selected TEP combinations (range: 0–100%). Only three patients did not present any of the semi-personalised TEP combinations.

Additionally, nine HLA class II TEP were identified covering 62.5% of the whole cohort (HPV-positive: 72.7%; HPV-negative: 50.0%) as shown in Fig. 5d.

With the described HLA class I and II TEP selection, a warehouse was established covering all OPSCC patients and offering potential target peptides for immunotherapeutic approaches in the course of OPSCC treatment.

## Discussion

Here, we present the first comprehensive analysis of the immunopeptidome in an OPSCC cohort with a representative distribution of HLA allotypes for the German population. Interestingly, HLA-B*51:01 was significantly overrepresented in the OPSCC cohort compared to a reference cohort. We identified OPSCC-exclusive class I and class II peptides in each patient sample including peptides shared by several patients with the respective HLA allele. In total, >5000 OPSCC-exclusive class I ligands and 5468 HLA class II-presented peptides respectively were discovered, some of which were newly identified. A peptide warehouse containing tumour-exclusive peptides (TEP) was generated containing potential targets for immunotherapy of OPSCC. None of the selected TEP were derived from previously germline antigens, commonly overexpressed proteins, or oncogenes. Instead, many TEP were derived from matrix proteins such as collagens, keratins, fibronectin or plakophilin.

The respective significance of the different types of cancer antigens for immunotherapy is currently unclear. An interesting finding in two patients with cervical cancer treated with adoptive T cell transfer with T cells primed for viral, HPV-specific antigens, challenges the central role of viral antigens even for HPV-associated disease [45]. Interestingly, the main portion of transfused immune cells was not directed against the viral antigens that were used for stimulation and expansion, but against a non-mutated germline associated antigen or a mutation-associated neoantigen, respectively. Thus, viral, mutational and non-mutated cancer antigens may play a role in the recognition of OPSCC.

However in this analysis, none of the TEP was derived from an HPV protein or a predicted, mutated neoantigen although HPV-specific peptides and individual mutated neoantigens were specifically queried in the dataset. This may be due to a lower presentation level of peptides from such antigens in comparison to other TEP. We cannot rule out that such peptides are presented at levels undetectable with the sensitivity of MS applied in this study. However, if they are presented, they are presented at much lower levels than other TEP. No significant differences were found between sample mass, the number of total peptides or TEP presented by HPV status. These results indicate that the failure to detect HPV peptides cannot be attributed to virally induced reduction of HLA molecules on the tumour [46–48]. Nevertheless, HPV-associated molecular differences like genetic, epigenetic or transcriptomic alterations were also mirrored in the HLA ligandome resulting in clustering of patient ligandome samples according to HPV status.

Still, HPV-specific T cell immunity has been detected in HPV-associated cancers and is associated with improved prognosis [17, 18, 49–51]. Also, HPV-specific vaccines have been successful in early clinical trials [52–55]. Thus, the integration of HPV-specific antigens into a semi-personalised or personalised multiantigen vaccine seems rational due to the high immunogenicity of viral antigens.

Another open question is the optimal strategy for antigen selection in vaccination trials: Should patients be selected based on the presence of a certain antigen (antigen-dependent enrolment) or are personalised, custom-manufactured vaccines needed for each patient? Is there a role for multiantigen vaccines developed for semi-personalised vaccination (i.e. an antigen selection based upon the HLA type of the patient) and statistically covering untested patients with at least one of the selected antigens?

The number of individual and shared TEP identified is promising and allows for both, a personalised and a semi-personalised multiantigen vaccination strategy. The strategy described here, is the definition and production of a warehouse of HLA-specific peptide combinations covering a high proportion of untested patients with the respective HLA type combined by a TEP selection for HLA class II, both based on multiobjective optimisation [41]. After immunogenicity testing, from this warehouse, a semi-personalised vaccine will be tested composed of the selection of peptide combinations for the individual patient’s HLA type preferably in combination with an HPV vaccine in HPV-positive patients. This strategy may reach a coverage comparable with a personalised neoepitope vaccine [16], avoiding the time and cost for individual analysis of the patient’s tumour mutanome and manufacturing of a personalised vaccine de novo.

Many past and ongoing trials in head and neck cancer focus on neoadjuvant immunotherapy achieving a pathologically confirmed immune response in a high fraction of patients [14, 56–61]. The semi-personalised strategy makes neoadjuvant immune checkpoint modulation combined with vaccination possible, which may further increase response rates. The tumour material harvested during surgery could potentially be used to optimise target antigen selection for an adjuvant phase of immunotherapy. A similar approach has been successfully employed in glioblastoma using a warehouse-based HLA-adapted vaccine followed by personalised vaccination [62].

In conclusion, the immunopeptidome of OPSCC differs by HPV status although we found no HPV-specific peptides. Instead, a number of TEP, some of which were found repeatedly, was identified and was used to build a peptide warehouse for semi-personalised vaccination as an addition to OPSCC immunotherapy. A final validation of immunogenicity of the warehouse peptides is needed before clinical application.

## Supplementary information

Supplementary Table 1

Supplementary Figures and Tables

## Data Availability

The MS raw data have been deposited to the ProteomeXchange Consortium (http://proteomecentral.proteomexchange.org) via the PRIDE partner repository (Perez-Riverol et al. [63]) with the dataset identifier PXD033383. WES data are available at the the European Genome-Phenome Archive (EGAS00001006477).
