# Supplementary material for: The HLA ligandome of oropharyngeal squamous cell carcinomas reveals shared tumour-exclusive peptides for semi-personalised vaccination
Source: Br J Cancer. 2023 Feb 23;128(9):1777–87. doi: 10.1038/s41416-023-02197-y (PMC9949688; doi:10.1038/s41416-023-02197-y)
Supplement: Supplementary file 2 — Supplementary Figures and Tables [file 41416_2023_2197_MOESM2_ESM.docx]

.
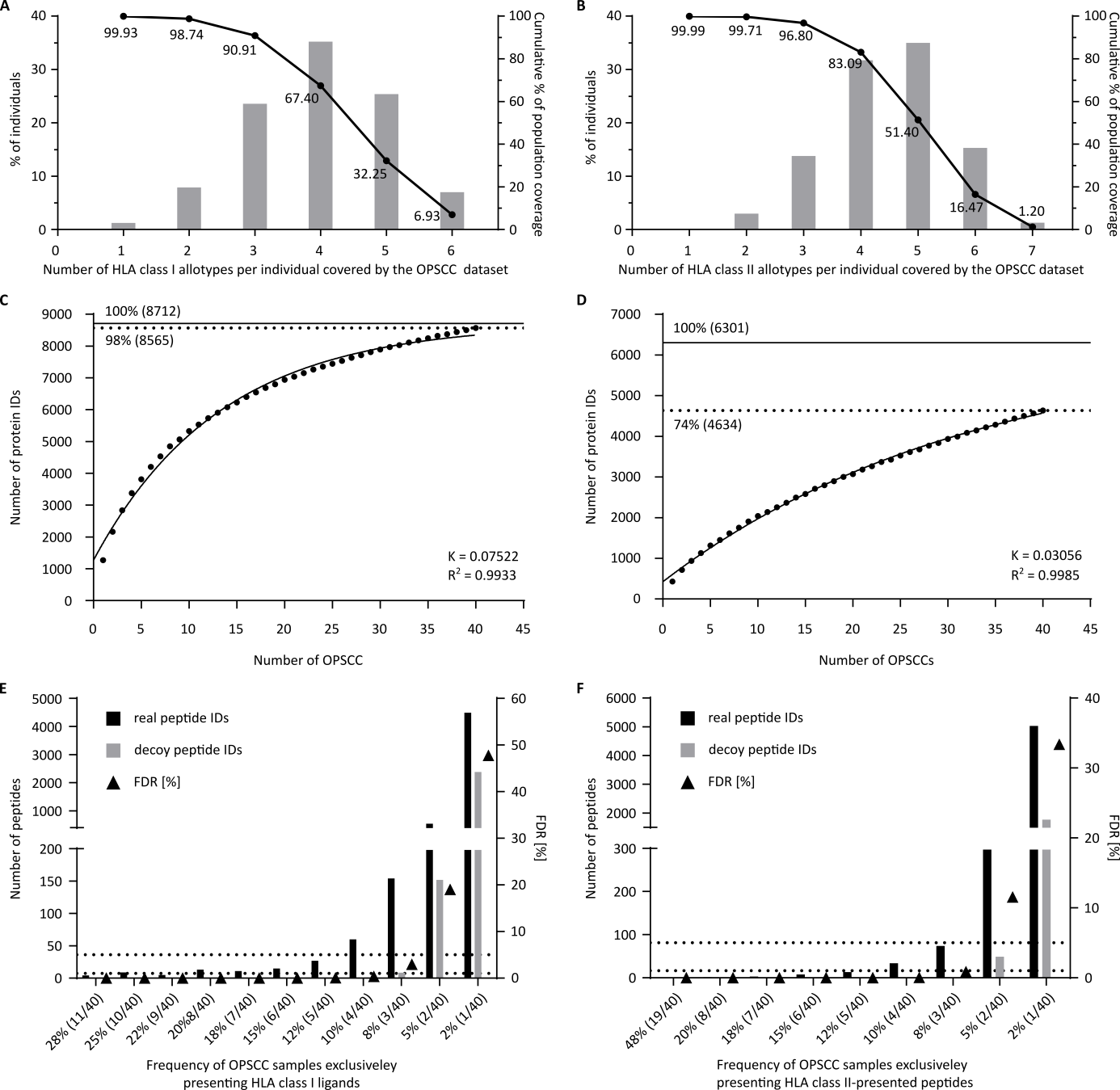


**Supplementary Figure 1:** **(A)** HLA class I and **(B)** HLA class II allotype population coverage within the OPSCC patient cohort compared to the world population (calculated by the IEDB population coverage tool, www.iedb.org) [1]. The frequencies of individuals within the world population carrying up to six HLA allotypes (x-axis) of the respective CML or benign dataset are indicated as grey bars on the left y-axis. The cumulative percentage of population coverage is depicted as black dots on the right y-axis. **(C)** Saturation analysis of HLA class I ligand source proteins and **(D)** the source proteins of HLA class II-presented peptides of the OPSCC patient cohort. Number of unique source protein identifications are shown as a function of cumulative HLA ligandome analysis of OPSCC samples (n = 40). Exponential regression allowed for the robust calculation (R^2^ = 0.9933 and R = 0.9985) of the maximum attainable number of different source protein identifications (black line). The dashed line depicts the source proteome coverage achieved in the OPSCC patient cohort. **(E)** HLA class I and **(F)** HLA class II peptide presentation were calculated for different presentation frequencies. The process of peptide randomization, cohort assembly and tumor‐associated peptide identification was repeated 1,000 times and the mean value of resulting decoy identifications was calculated and plotted for the different threshold values together with the real peptide identifications. The corresponding FDRs for any chosen tumor‐associated peptide threshold are listed below the x‐axis. OPSCC – oropharyngeal squamous cell carcinoma; n(OPSCCs) = 40; FDR – false discovery rate.


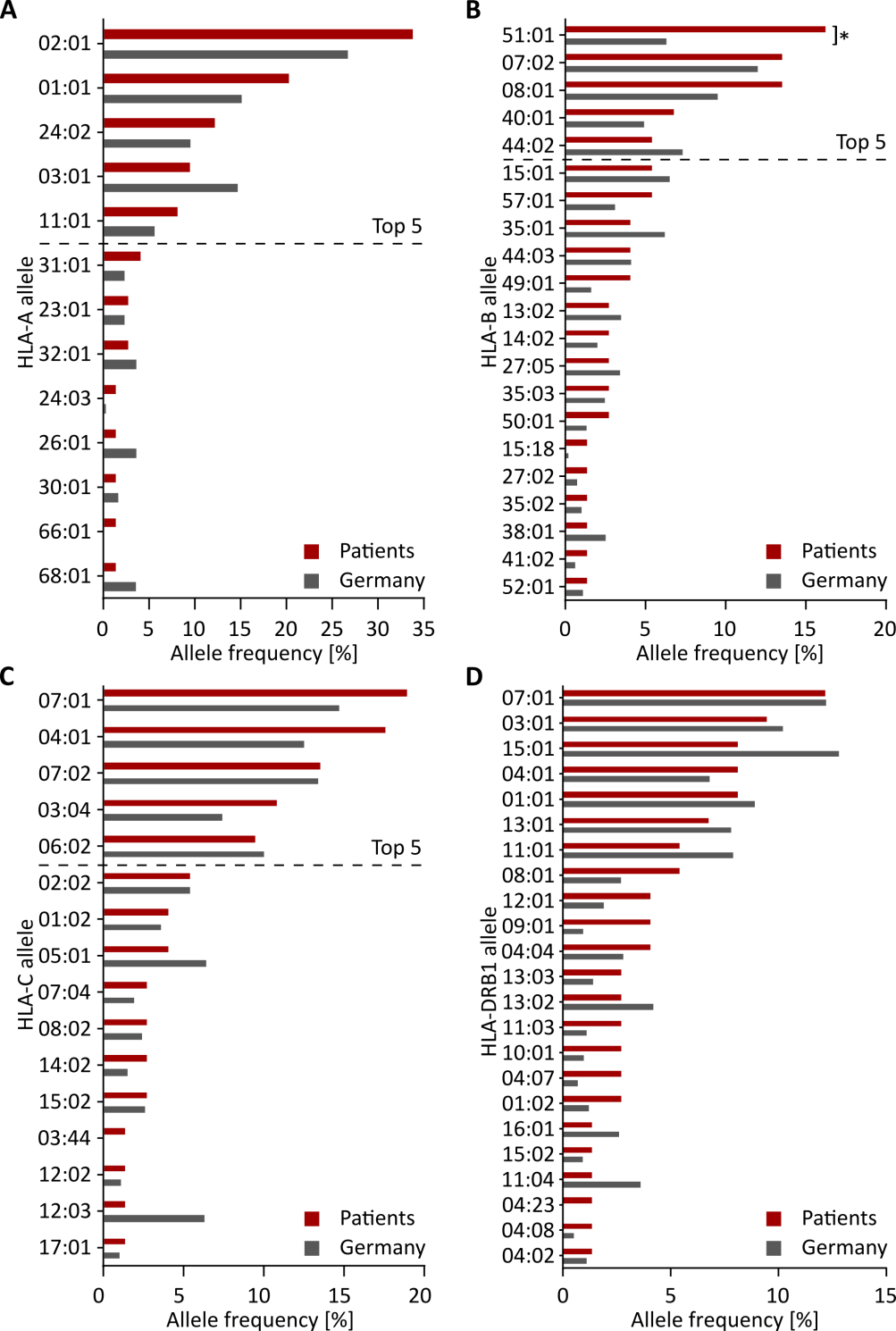


**Supplementary Figure 2: (A)** HLA-A, **(B)** HLA-B, **(C)** HLA-C and **(D)** HLA-DRB1 allelic distribution in the OPSCC patient cohort and a German reference population (“Germany pop 8”, Allele Frequency Net Database AFND, www.allelefrequencies.net) [2]. Top 5 assigns the five most frequent alleles within the patient cohort. OPSCC – oropharyngeal squamous cell carcinoma; n(Patients) = 40; n(Germany) = 39,689; * – p-value ≤ 0.05 after Bonferroni correction.


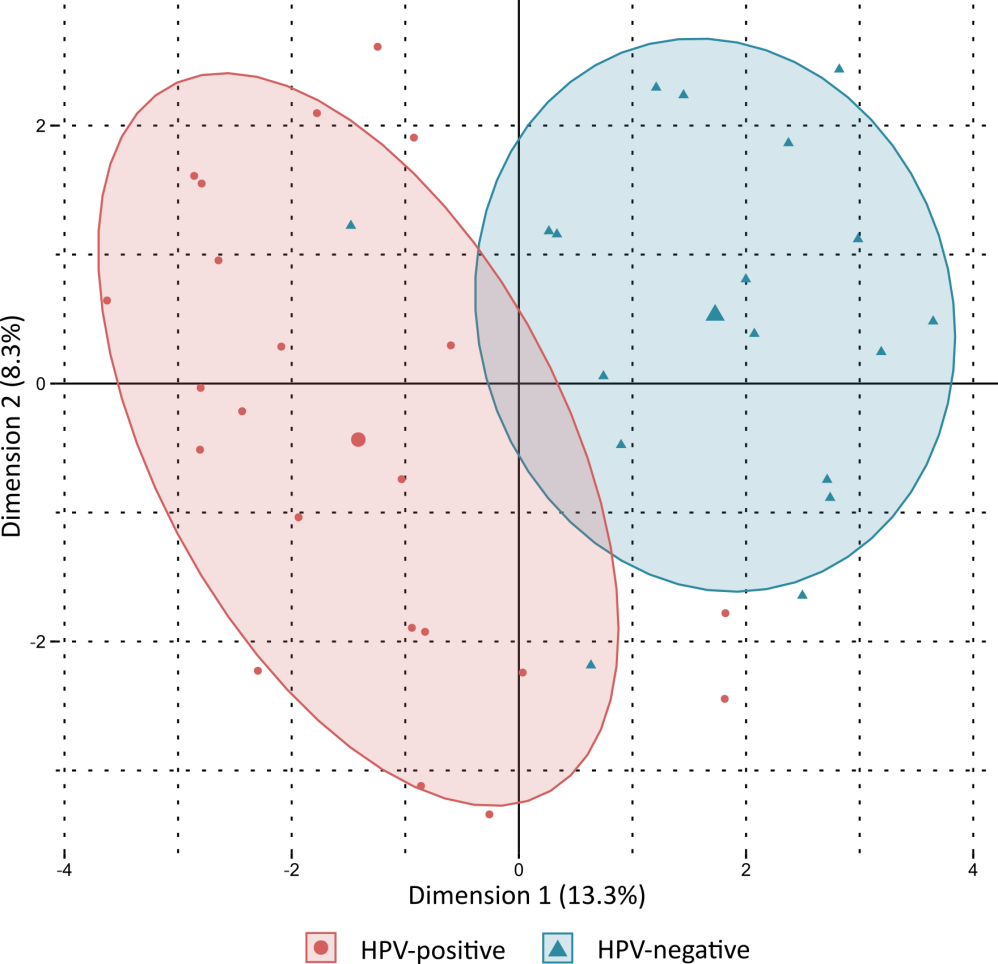


**Supplementary Figure 3:** Scatter plot of the supervised PCA on the basis of HLA ligands identified in HPV⁺ and HPV⁻ OPSCCs. The PCA was based on the merged source proteins of HLA class I binders and HLA class II-presented peptides from OPSCC samples. n(HPV-positive samples) = 22; n(HPV-negative samples) = 18; n(high-impact proteins) = 190; PCA = principle component analysis.


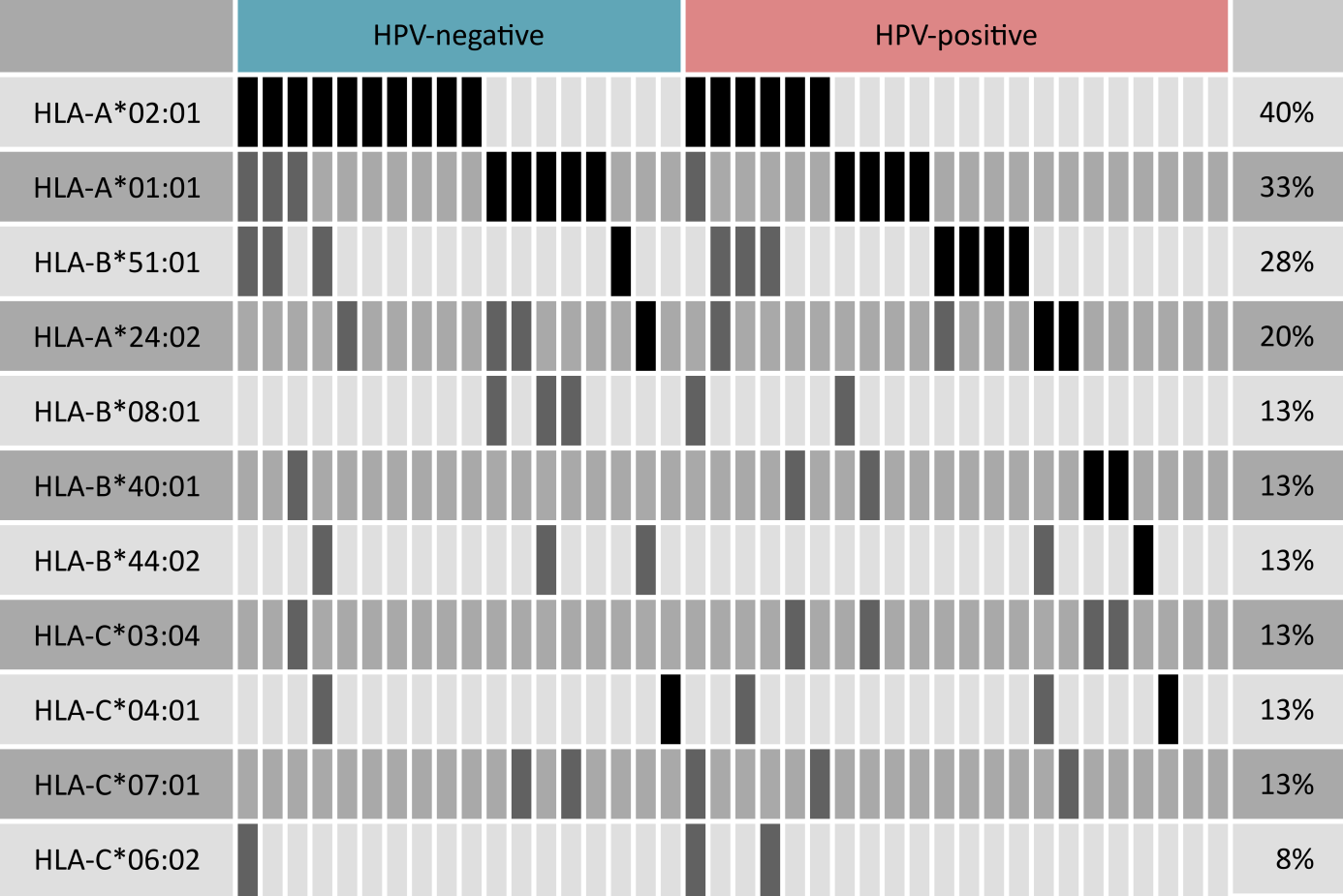


**Supplementary Figure 4:**

Coverage and overlap of the HLA-specific peptide selections by HPV-status. The peptide selections for the respective HLA-alleles were merged and are displayed in rows and individual patients in columns. The coverage for the respective peptide selection is indicated in the last column. Using all 29 peptides, all HPV-negative patients and 90.9% of HPV-positive patients were covered. Overlap was 1.167 for HPV-negative patients and 1.0 for HPV-positive patients.

**Supplementary Figure 5:**

Coverage and overlap of the HLA-class II peptide selections by HPV-status. Peptides are shown in rows and individual patients in columns. The coverage for the respective peptide selection is indicated in the last column. Using all 9 peptides, 62.5% of all patients (HPV-negative: 50%, HPV-positive: 72.7%) were covered.

Supplementary Table 1: HPV RNA read counts by HPV peptide and genotype.

Table provided in a separate file.

Supplementary Table 2: Overview of HLA ligandomic yields from OPSCCs. Isolated peptides were analyzed by LC-MS/MS. The HLA class I purity was calculated by the percentage of HLA class I ligands among the total number of HLA class I-presented peptides. The results of two biological replicates were merged for sample #26 and #27.

| **Sample number** | **Tissue mass [mg]** | **HLA class I** | | | | | **HLA class II** | |
| --- | --- | --- | --- | --- | --- | --- | --- | --- |
|  |  | **Presented peptides** | **Source proteins** | **Binders** | **Source proteins of binders** | **Purity [%]** | **Presented peptides** | **Source proteins** |
| **1** | 389 | 1253 | 1312 | 1192 | 1269 | 95 | 1050 | 600 |
| **2** | 326 | 1307 | 1362 | 1246 | 1310 | 95 | 574 | 391 |
| **3** | 132 | 1471 | 1481 | 1430 | 1446 | 97 | 820 | 473 |
| **4** | 371 | 2637 | 2311 | 2481 | 2204 | 94 | 1644 | 842 |
| **5** | 474 | 2092 | 1892 | 2019 | 1831 | 97 | 2086 | 920 |
| **6** | 303 | 1553 | 1502 | 1403 | 1386 | 90 | 1308 | 577 |
| **7** | 67 | 425 | 469 | 378 | 426 | 89 | 624 | 371 |
| **8** | 38 | 732 | 841 | 701 | 815 | 96 | 494 | 348 |
| **9** | 151 | 1059 | 1142 | 1025 | 1098 | 97 | 758 | 441 |
| **10** | 57 | 1578 | 1507 | 1468 | 1414 | 93 | 255 | 232 |
| **11** | 91 | 1336 | 1291 | 1200 | 1226 | 90 | 777 | 472 |
| **12** | 94 | 488 | 493 | 464 | 473 | 95 | 656 | 408 |
| **13** | 23 | 682 | 795 | 626 | 737 | 92 | 366 | 286 |
| **14** | 101 | 1431 | 1426 | 1355 | 1362 | 95 | 262 | 244 |
| **15** | 133 | 2452 | 2173 | 2330 | 2088 | 95 | 965 | 466 |
|  | 105 | 2067 | 1920 | 1962 | 1831 | 95 | 537 | 290 |
| **16** | 267 | 2494 | 2181 | 2400 | 2106 | 96 | 257 | 219 |
|  | 38 | 1166 | 1132 | 1099 | 1076 | 94 | 277 | 233 |
| **17** | 236 | 2282 | 2024 | 2143 | 1917 | 94 | 675 | 419 |
| **18** | 84 | 827 | 892 | 798 | 865 | 96 | 717 | 383 |
| **19** | 218 | 2442 | 2253 | 2358 | 2171 | 97 | 605 | 446 |
| **20** | 182 | 1964 | 1808 | 1876 | 1736 | 96 | 704 | 447 |
| **21** | 567 | 2243 | 2067 | 2127 | 1979 | 95 | 765 | 446 |
| **22** | 128 | 1354 | 1269 | 1288 | 1259 | 95 | 447 | 284 |
| **24** | 313 | 1770 | 1730 | 1683 | 1658 | 95 | 1424 | 706 |
| **25** | 278 | 1452 | 1431 | 1055 | 1091 | 73 | 871 | 959 |
| **26** | 159 | 1753 | 1788 | 1662 | 1603 | 95 | 830 | 490 |
| **27** | 206 | 1683 | 1638 | 1493 | 1529 | 89 | 802 | 434 |
| **28** | 203 | 1520 | 1457 | 1463 | 1411 | 96 | 901 | 458 |
| **29** | 37 | 1367 | 1360 | 1303 | 1297 | 95 | 438 | 279 |
| **30** | 35 | 330 | 371 | 308 | 347 | 93 | 168 | 154 |
| **31** | 17 | 302 | 380 | 265 | 338 | 88 | 178 | 149 |
| **32** | 80 | 1673 | 1649 | 1616 | 1597 | 97 | 957 | 517 |
| **33** | 80 | 567 | 656 | 528 | 636 | 93 | 224 | 148 |
| **34** | 57 | 1445 | 1437 | 1331 | 1348 | 92 | 341 | 301 |
| **35** | 107 | 1201 | 1256 | 1148 | 1199 | 96 | 177 | 193 |
| **36** | 85 | 555 | 609 | 514 | 566 | 93 | 201 | 139 |
| **37** | 87 | 803 | 866 | 776 | 845 | 97 | 242 | 163 |
| **38** | 252 | 1057 | 1123 | 1012 | 1081 | 96 | 767 | 432 |
| **39** | 333 | 1627 | 1466 | 1551 | 1390 | 95 | 1038 | 611 |
| **40** | 177 | 2083 | 1808 | 2023 | 1875 | 97 | 1079 | 447 |

Supplementary Table 3: Overview of HLA ligandomic yields from tonsils. Isolated peptides were analyzed by LC-MS/MS. The HLA class I purity was calculated by the percentage of HLA class I ligands among the total number of HLA class I-presented peptides. T = tonsil.

| **Sample number** | **Tissue mass [mg]** | **HLA class I** | | | | | **HLA class II** | |
| --- | --- | --- | --- | --- | --- | --- | --- | --- |
|  |  | **Presented peptides** | **Source proteins** | **Binders** | **Source proteins of binders** | **Purity [%]** | **Presented peptides** | **Source proteins** |
| **T1** | 378 | 2247 | 2097 | 2144 | 2006 | 95 | 1614 | 770 |
| **T2** | 302 | 2728 | 2366 | 2586 | 2272 | 95 | 1026 | 559 |
| **T3** | 210 | 1670 | 1635 | 1602 | 1579 | 96 | 1509 | 649 |
| **T4** | 487 | 2610 | 2350 | 2463 | 2229 | 94 | 2012 | 900 |
| **T5** | 162 | 1685 | 1599 | 1615 | 1542 | 96 | 1044 | 524 |

**Supplementary Table 4:** **Key data of the optimization process using AVAtar** [3]. TEP – tumor-exclusive peptide, pt – patient, QC – quality control.

| **HLA-allele** | **HLA-allotype** | **TEP#** | **TEP# in**  **≥ 2 pt** | **TEP candidates** | **failed QC** | **TEP selected** | **Coverage of selection** | **Overlap of selection** |
| --- | --- | --- | --- | --- | --- | --- | --- | --- |
| HLA-A | *02:01 | 367 | 57 | 15 | 5 | 7 | 0.762 | 0.667 |
|  | *01:01 | 247 | 27 | 8 | 1 | 4 | 0.929 | 0.786 |
|  | *24:02 | 121 | 23 | 5 | 2 | 2 | 0.778 | 0.333 |
|  | *11:01 | 43 | 2 | 2 | 1 | 0 | n.a. | n.a. |
|  | *03:01 | 76 | 3 | 3 | 2 | 0 | n.a. | n.a. |
| HLA-B | *51:01 | 184 | 48 | 3 | 0 | 3 | 0.833 | 0.250 |
|  | *07:02 | 95 | 6 | 4 | 2 | 0 | n.a. | n.a. |
|  | *08:01 | 128 | 20 | 6 | 2 | 2 | 0.500 | 0.100 |
|  | *44:02 | 321 | 45 | 3 | 0 | 1 | 0.500 | 0.000 |
|  | *40:01 | 95 | 37 | 6 | 1 | 2 | 1.000 | 0.200 |
| HLA-C | *07:01 | 109 | 6 | 4 | 2 | 2 | 0.385 | 0.000 |
|  | *07:02 | 148 | 3 | 1 | 0 | 0 | n.a. | n.a. |
|  | *04:01 | 199 | 9 | 2 | 0 | 2 | 0.455 | 0.000 |
|  | *03:04 | 264 | 10 | 6 | 3 | 3 | 0.625 | 0.250 |
|  | *06:02 | 26 | 2 | 1 | 0 | 1 | 0.429 | 0.000 |

**Supplementary Table 5:** OPSCC warehouse of selected HLA class I ligands. AC – accession, GN – gene name.

| **Peptide** | | | | **Source protein** | | |
| --- | --- | --- | --- | --- | --- | --- |
| **HLA restriction** | **Sequence** | **Representation frequency in** | | **UniProtKB AC** | **GN** | **Protein name** |
|  |  | **cohort** | **allotype-positive samples** |  |  |  |
| **A*02:01** | **ALLGSAFQL** | 8% | 14% | Q96MG2 | JSRP1 | Junctional sarcoplasmic reticulum protein 1 |
|  | **ALTDIVSQV** | 10% | 18% | Q9NQU5 | PAK6 | Serine/threonine-protein kinase PAK 6 |
|  | **GLWEDGRSTLL** | 8% | 14% | Q96BA8 | CREB3L1 | Cyclic AMP-responsive element-binding protein 3-like protein 1 |
|  | **GVLENIFGV** | 13% | 23% | Q9H6A9 | PCNX3 | Pecanex-like protein 3 |
|  | **KLAEISLGV** | 10% | 18% | P32926 | DSG3 | Desmoglein-3 |
|  | **RLDDLKMTV** | 13% | 23% | Q13753 | LAMC2 | Laminin subunit gamma-2 |
|  | **RLLEGEDAHL** | 15% | 27% | P02533;P08779;Q04695 | KRT14;16;17 | Keratin, type I cytoskeletal 14;16;17 |
| **A*01:01** | **EMEAQNQEY** | 10% | 29% | P19012 | KRT15 | Keratin, type I cytoskeletal 15 |
|  | **RTDIARTEY** | 10% | 29% | Q14204 | DYNC1H1 | Cytoplasmic dynein 1 heavy chain 1 |
|  | **RTEFNLNQY** | 20% | 57% | Q99715 | COL12A1 | Collagen alpha-1(XII) chain |
|  | **YSELASHVVSY** | 20% | 57% | Q9UMD9 | COL17A1 | Collagen alpha-1(XVII) chain |
| **C*07:01** | **YTFRYPLSL** | 8% | 23% | P24347 | MMP11 | Stromelysin-3 |
|  | **ARLAFVIVF** | 5% | 15% | Q5XXA6 | ANO1 | Anoctamin-1 |
| **B*51:01**  **(B*52:01)** | **DATETTITI** | 8% | 23% | P02751 | FN1 | Fibronectin |
|  | **DQYKFLAV** | 18% | 54% | Q9NRX3 | NDUFA4L2 | NADH dehydrogenase [ubiquinone] 1 alpha subcomplex subunit 4-like 2 |
|  | **VALPVYLLI** | 10% | 31% | P57054 | PIGP | Phosphatidylinositol N-acetylglucosaminyltransferase subunit P |
| **C*04:01** | **SPEDGIHEL** | 8% | 25% | P02751 | FN1 | Fibronectin |
|  | **VFDLVELEVL** | 5% | 17% | Q8IWT6 | LRRC8A | Leucine-rich repeat-containing protein 8A |
| **B*08:01** | **HGTIKNQL** | 8% | 30% | Q86XI2 | NCAPG2 | Condensin-2 complex subunit G2 |
|  | **VAAPRWVL** | 8% | 30% | A6NJ16 | IGHV4OR15-8 | Putative V-set and immunoglobulin domain-containing-like protein IGHV4OR15-8 |
| **A*24:02**  **(A*23:01)** | **KYMYFTVVM** | 18% | 58% | Q8WV24 | PHLDA1 | Pleckstrin homology-like domain family A member 1 |
|  | **NWPSRPYLF** | 10% | 33% | O43795 | MYO1B | Unconventional myosin-Ib |
| **C*03:04** | **SAVKEGTAM** | 5% | 22% | Q9H694 | BICC1 | Protein bicaudal C homolog 1 |
| **B*44:02**  **(B*44:03)** | **EETNPKGSGW** | 13% | 64% | Q13835 | PKP1 | Plakophilin-1 |
| **B*40:01** | **REVVDPEVFF** | 8% | 50% | Q13835 | PKP1 | Plakophilin-1 |
|  | **SEPNDVFFKL** | 8% | 50% | P12111 | COL6A3 | Collagen alpha-3(VI) chain |
| **C*03:04** | **YEEAMKEVL** | 5% | 22% | Q9P0K7 | RAI14 | Ankycorbin |
|  | **YEIDDVERL** | 8% | 33% | Q9ULI4;Q2KJY2 | KIF26A;B | Kinesin-like protein KIF26A;B |
| **C*06:02** | **ARLIPIIVL** | 8% | 43% | B0I1T2 | MYO1G | Unconventional myosin-Ig |
| **HLA-Class II** | **IRQFTSSSSIKGSSG** | 18% | n.a. | Q04695 | KRT17 | Keratin, type I cytoskeletal 17 |
|  | **LTSTSGPGFHLMLPFI** | 13% |  | O94905 | ERLIN2 | Erlin-2 |
|  | **ELKKKLKEKAKERREKEMLERLEK** | 10% |  | Q9UIG0 | BAZ1B | Tyrosine-protein kinase BAZ1B |
|  | **ENDVIISINGQSVVSA** | 10% |  | Q92743 | HTRA1 | Serine protease HTRA1 |
|  | **IQDFIEAEDDLSSFR** | 10% |  | Q15063 | POSTN | Periostin |
|  | **PVHWQFGQLDQHPIDG** | 8% |  | P09486 | SPARC | SPARC |
|  | **FADFTFATGKIIG** | 8% |  | Q13093 | PLA2G7 | Platelet-activating factor acetylhydrolase |
|  | **IKNIISEYKSAIQSQKRRRPRYRKR** | 8% |  | Q6RI45 | BRWD3 | Bromodomain and WD repeat-containing protein 3 |
|  | **VDEEEVKFPGTNFDE** | 8% |  | Q9BVV6 | KIAA0586 | Protein TALPID3 |

References

[1] Bui H-H, Sidney J, Dinh K, Southwood S, Newman M J, Sette A. Predicting population coverage of T-cell epitope-based diagnostics and vaccines. *BMC Bioinformatics* 7 , 153 (2006).

[2] Gonzalez-Galarza F F, McCabe A, Santos E J M D, Jones J, Takeshita L, Ortega-Rivera N D, Del Cid-Pavon G M, Ramsbottom K, Ghattaoraya G, Alfirevic A, Middleton D, Jones A R. Allele frequency net database (AFND) 2020 update: gold-standard data classification, open access genotype data and new query tools. *Nucleic Acids Res* 48 (D1), D783-D788 (2020).

[3] Völkel G, Laban S, Fürstberger A, Kühlwein S D, Ikonomi N, Hoffman T K, Brunner C, Neuberg D S, Gaidzik V, Döhner H, Kraus J M, Kestler H A. Analysis, identification and visualization of subgroups in genomics. *Brief Bioinform* 22 (3) (2021).
